# Genital and Intertriginous Rashes Refractory to Antimicrobial Treatments: Have You Thought about Crohn's Disease?

**DOI:** 10.1155/2021/5578810

**Published:** 2021-07-23

**Authors:** Candelaria O'Farrell, Annette Roberts, Claudia Riera Canales, Carrie Firestone Baum, Lina Maria Felipez

**Affiliations:** Nicklaus Children's Hospital, Miami, FL, USA

## Abstract

Crohn's disease is an inflammatory bowel disease that can have multiple extraintestinal manifestations and can develop prior to, following, or simultaneously with gastrointestinal tract involvement (Aberumand et al. (2017), Georgious et al. (2006), Larsen et al. (2010), Levine and Burakoff (2011), Louis et al. (2018)). This report examines the case of a 16-year-old male with a rash of the genital, intergluteal, and inguinal regions refractory to antimicrobial treatments suspicious for an extraintestinal manifestation of Crohn's disease. The patient was diagnosed with inflammatory, nonfistulizing colonic Crohn's disease following presentation with gastrointestinal symptoms including abdominal pain and bloody stools 6 months after the onset of the rash. The genital lesions resolved after starting treatment for Crohn's disease with adalimumab.

## 1. Introduction

Crohn's disease (CD) is a type of inflammatory bowel disease that can present with extraintestinal manifestations and can develop prior to, following, or simultaneously with gastrointestinal tract involvement and symptoms [1–5]. These extraintestinal manifestations may present as cutaneous lesions of the genital region. Rarely, extraintestinal manifestations of Crohn's disease can manifest as cutaneous noncaseating granulomas outside of the gastrointestinal tract and is referred to as metastatic Crohn's disease [1, 5–9]. Cutaneous manifestations of CD may occasionally precede intestinal involvement and can occur anywhere on the body including the genital regions [2–7]. Anti-TNF medications have been used in the management of Crohn's disease with improvement of extraintestinal manifestations of Crohn's [7, 10].

## 2. Case Presentation

**Figure 1:**
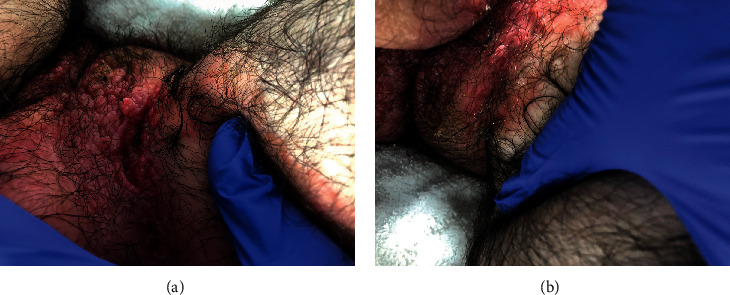
Well-demarcated erythematous plaques, nodules, and papules noted on the groin (a) and gluteal cleft (b) observed prior to treatment with adalimumab.

**Figure 2:**
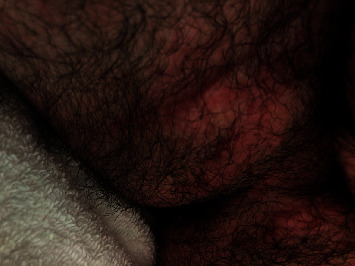
Improvement following 8 months of treatment with adalimumab.

Our patient, a 16-year-old male, initially presented for evaluation of abdominal pain, bloody stools, and weight loss. 6 months prior to the onset of his abdominal symptoms, he had developed a nonpruriginous, painless, erythematous, scaly, and raised rash on his inguinal, scrotal, and intergluteal regions (Figure 1). He had no other notable past medical history. He was originally diagnosed with jock's itch and treated with topical ciclopirox without improvement. After failing treatment with topical ciclopirox, he was treated for suspected bacterial and fungal infections with PO doxycycline and topical mupirocin and ketoconazole creams also without improvement. Dermatophyte test medium (DTM) fungal cultures were negative, and an aerobic culture grew *Staphylococcus* and *Moraxella catarrhalis*. Aluminum acetate soaks were added with only partial improvement of rash. Upon reexamination, he was noted to have well-demarcated erythematous scaly plaques on the groin folds and gluteal cleft with associated skin-colored nodules and papules, as well as erythema and edema of the scrotum. Meanwhile, he underwent endoscopy, colonoscopy, and MRI enterography for workup of his abdominal symptoms. His endoscopy was unremarkable. Colonoscopy visualized congestion, erosions, friability, and loss of vascularity consistent with moderate inflammation in the rectum and sigmoid colon. Biopsies showed chronic inflammatory changes with focal epithelioid granulomata in the colon with negative acid-fast bacilli and fungal stains. MRI enterography noted bowel thickening with surrounding inflammation extending from the colonic splenic flexure to the rectum. A diagnosis of inflammatory, nonfistulizing colonic CD was made. After 2 weeks of topical fluocinolone twice daily and induction therapy with adalimumab followed by subsequent doses of 40 mg every other week, the rash had greatly improved, consistent with an extraintestinal manifestation of his CD (Figure 2). His adalimumab dose was increased to 40 mg weekly due to subtherapeutic drug levels (6.3 ug/ml) and persistence of GI symptoms. Continued improvement of the rash was noted with the subsequent dose increase.

## 3. Discussion

**Figure 3:**
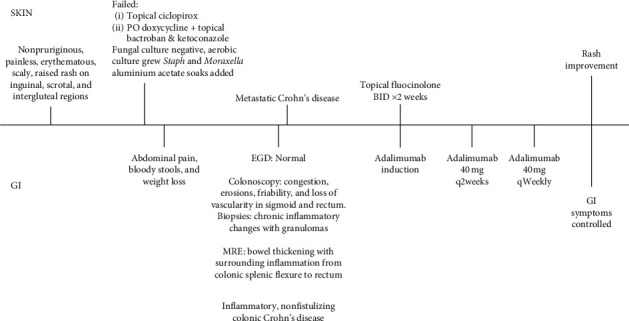
Timeline depicting the series of events occurring from the onset of the rash to its resolution following appropriate therapy.

CD is a multisystemic chronic inflammatory disorder that can present with cutaneous findings preceding gastrointestinal symptoms of CD such as abdominal pain and bloody stools. Early recognition of extraintestinal cutaneous manifestations of CD can lead to a timely diagnosis and treatment of CD with resolution of both intestinal and extraintestinal symptoms. Thus, when encountering genital or intertriginous rashes refractory to antimicrobial treatments, underlying inflammatory bowel disease should be investigated (Figure 3).

## Data Availability

No data were used to support the findings of this study.
